# Characteristics of Perpetrators of Neonaticide

**DOI:** 10.1192/j.eurpsy.2022.92

**Published:** 2022-09-01

**Authors:** C. Klier, S. Amon, P. Fernandez Arias

**Affiliations:** 1 University of Vienna, Department Of Pediatrics And Adolescent Medicine, Wien, Austria; 2 Barmherzige Brüder, Inquisitenabteilung, Vienna, Austria; 3 Monash Deakin Filicide Research Hub, Social Work, Melbourne, Australia

**Keywords:** neonaticide

## Abstract

Authors: Klier CM, Amon S, Fernandez Arias P Introduction Denial of pregnancy the most important risk factor for neonaticide and is a frequent pregnancy associated disorders. It occurs more often than placenta previa or uterus rupture. Wessel found that 1 in 475 women deny pregnancy, but a new study by Simermann even found a higher prevalence of 1:300. We studied how women face their reproductive potential before the denied pregnancy occurs. Moreover pregnancy variables in this group were compared to Austrian population data. Methods In total, there were 69 neonaticide victims between 1995 and 2017. We analysed 55 neonaticide cases, committed by 48 perpetrators, whereas 4 of them were repeated perpetrator and responsible for 11 cases. We also included three cases of suspected neonaticides, whereas later the coroner identified a natural cause of death. The datasheet contains 553 variables, all cases were code by one rater SA. Specifically we looked at the following variables: method of contraception, partner’s interest in contraception, abortion, evidence of pregnancy, motives of pregnancy negation, pregnancy symptoms, prenatal care, awareness of environment, living situation during pregnancy and at birth, immediate reaction to birth Results We found a high percentage of women who did not use contraception (53%), specifically when compared to the percentages of the Austrian population data from a recent survey (26%). When the contraceptive methods were looked at, women in the neonaticide group did practically not use any method with a pearl index of <4, in comparison to 20% in the general population. A high adolescent birth rate (47%) could be shown in comparison to the population (3%). Conclusions It needs further research to find out how denial of pregnancy and reproductive potential are connected and if the reasons for denial of pregnancy are the precursors of the reproductive denial also.

**Disclosure:**

No significant relationships.

